# Gasdermin E: A Prospective Target for Therapy of Diseases

**DOI:** 10.3389/fphar.2022.855828

**Published:** 2022-04-06

**Authors:** Xiu-Xiu Liao, Yong-Zhao Dai, Yao-Zhong Zhao, Ke Nie

**Affiliations:** School of Chinese Materia Medica, Guangdong Pharmaceutical University, Guangzhou, China

**Keywords:** gasdermin E, programmed cell death, target therapy, review, diseases

## Abstract

Gasdermin E (GSDME) is a member of the gasdermin protein family, which mediates programmed cell death including apoptosis and pyroptosis. Recently, it was suggested that GSDME is activated by chemotherapeutic drugs to stimulate pyroptosis of cancer cells and trigger anti-tumor immunity, which is identified as a tumor suppressor. However, GSDME-mediated pyroptosis contributes to normal tissue damage, leading to pathological inflammations. Inhibiting GSDME-mediated pyroptosis might be a potential target in ameliorating inflammatory diseases. Therefore, targeting GSDME is a promising option for the treatment of diseases in the future. In this review, we introduce the roles of GSDME-driven programmed cell death in different diseases and the potential targeted therapies of GSDME, so as to provide a foundation for future research.

## Introduction

Gasdermin E (GSDME), located on human chromosome 7p15, is genetically encoded as a 496 amino acids protein with 10 exons (Van Laer et al., 1998). As the sequence and structure of GSDME are similar to the gasdermins, it was classified as a member of the gasdermin family in 2007, which consists of gasdermin A (GSDMA), gasdermin B (GSDMB), gasdermin C (GSDMC), gasdermin D (GSDMD), GSDME and Pejvakin (PJVK) (Tamura et al., 2007; Feng et al., 2018). There is evidence suggesting that GSDME regulates apoptosis and pyroptosis, the two forms of programmed cell death (PCD). (Lage et al., 2001; Op de Beeck et al., 2011; Rogers et al., 2017; Wang et al., 2017). The two domains in the GSDME protein, an autoinhibitory C-terminal domain and a cytotoxic N-terminal domain (De Schutter et al., 2021), maintain the stability or transformation of its conformation to exert the biological function of autoinhibitory and regulation of PCD.

The capacity of GSDME to induce cell death determines its critical role in diseases. The *gsdme* gene, previously referred to deafness autosomal dominant 5 (DFNA5), was initially found to be related to non-syndromic hearing loss, which is concerned with apoptosis (Van Camp et al., 1995; Van Laer et al., 1998; Op de Beeck et al., 2011). Another form of PCD, the pyroptosis driven by GSDME, has attracted attention in recent years. Upon cleavage by caspase-3 specifically, the N-terminal fragments of GSDME are generated to activate the pore-forming properties and ultimately trigger pyroptosis. (Rogers et al., 2017; Wang et al., 2017). GSDME, a tumor suppressor, has tremendous potential as a tumor biomarker, mediating inflammatory death of tumor cells and activating immune responses in favor of anti-tumor therapy (Wang et al., 2017; Zhang Z. et al., 2020). However, it is also responsible for pathological inflammations, contributing to disease developments. As a consequence of the discovery, the passive effects of chemotherapy were exacerbated by GSDME-induced pyroptosis, the role of GSDME in normal tissue destruction has been gradually realized (Wang et al., 2017), which implies a bright prospect of GSDME as a therapeutic target for diseases, not limited to cancers. In this review, we emphasize the PCD mediated by GSDME, summarize the key roles of GSDME in both cancer and non-cancer diseases, and describe the potential therapeutic approaches targeting GSDME, so as to provide directions for the targeted therapy in the future.

## GSDME-Mediated Programmed Cell Death

Apoptosis, pyroptosis and necroptosis are the best characterized forms of PCD, which facilitate the initiation and execution of cell death through its complex and diverse molecular mechanisms. Accumulated evidence suggests that GSDME-mediated PCD mainly involves apoptosis and pyroptosis, and that GSDME formerly functioned as an apoptosis-inducing protein before its identification as a pivotal executor of pyroptosis in 2017. Here, we examine the mechanisms of PCD mediated by GSDME and the crosstalk among them.

### The Apoptosis Related to GSDME

When GSDME was discovered as a gene associated with hearing loss, little was known about its physiological functions. Apoptosis-inducing property of GSDME was first discovered in early biological research, which was widely known as a non-inflammatory PCD to clear unwanted cells. Morphologically, apoptosis is characterized by contracted cells, condensed chromatin, fragmented DNA, and apoptotic bodies formation. As early as 2001, it was reported that melanoma clone cells transfected with *gsdme* cDNA displayed DNA fragmentation and the cleavage of caspase-3, indicating that GSDME might be connected with apoptotic pathways (Lage et al., 2001). Further research has revealed that GSDME, a target of the tumor suppressor p53, may be responsive to DNA damage as a mediator of p53-dependent apoptosis. Specifically, elevated expression of GSDME may inhibit the apoptosis induced by etoposide without p53. On the contrary, GSDME may contribute to the etoposide-induced DNA damage with the presence of p53 (Masuda et al., 2006).

Given the evidence above, GSDME may be part of the complex mechanisms of apoptosis, but its expression seems to be insufficient to cause apoptosis. A report demonstrated that the expression of GSDME mRNA was up-regulated in response to apoptosis induced by dexamethasone (Dex) and in connection with apoptosis sensitivity, though merely altering the level of GSDME mRNA expression was not sufficient to affect the Dex-dependent apoptosis (Webb et al., 2007). Surprisingly, mutated GSDME caused apoptosis in association with oxidative stress, mitochondrial damage, and endoplasmic reticulum stress (Van Rossom et al., 2012; Van Rossom et al., 2015). Besides, the apoptotic characteristics of GSDME were proved in both overexpression experiments and physiological environments (Op de Beeck et al., 2011). The apoptosis-inducing structure of GSDME further demonstrated that the physiological function of GSDME is intrinsically linked to apoptosis. Two critical domains, the C-terminal domain and the N-terminal domain associated with apoptosis, were identified. The C-terminal domain could shield the apoptosis-inducing ability of the N-terminal domain that possessed a complete region between exon 2 and exon 6 (Op de Beeck et al., 2011). It seems to explain why transfection of GSDME alone does not lead to apoptosis, whereas mutated GSDME does. The mutation site of GSDME is usually near the 8th exon of the C-terminal, which may lead to the disappearance of the shielding effect of the C-terminal and display apoptotic activity. In contrast, a study indicated that the hepatocellular carcinoma cells were prevented from proliferating when GSDME was overexpressed, and the underlying mechanism was proposed to be potentially linked to apoptosis due to an increased frequency of apoptosis and the increased expression of apoptotic proteins including Fas and caspase-8 (Wang et al., 2013). Another study also found that the lung cancer cells were promoted to undergo apoptosis by GSDME, in consistence with the finding described by Wang *et al.* (Kong, 2013). The differences in these studies have not been well explained, suggesting that the proposed role of GSDME in apoptosis should be revisited in light of its now well-established role as a switch that can turn apoptosis into pyroptosis (Wang et al., 2017).

### The Pyroptosis Mediated by GSDME

The pyroptosis, which previously was misinterpreted as apoptosis, first appeared in 2001 as a term to describe programmed cell death with pro-inflammatory properties (Cookson and Brennan, 2001). Unlike apoptosis, pyroptosis features pore-forming membranes, cell swelling, plasma membrane rupture, and release of inflammatory substances (He et al., 2015; Jorgensen and Miao, 2015). For a long time, pyroptotic cell death was considered as monocytes death mediated by caspase-1 (Bergsbaken et al., 2009), which mediates the maturation of interleukin-1β (IL-1β) and interleukin-18 (IL-18) (Fantuzzi and Dinarello, 1999). It was not until 2015 that GSDMD was found as a key substrate for pyroptosis, which could be cleaved by inflammatory caspases and the released N-terminal binds to membrane lipids to induce membrane perforation, destructing cell osmotic pressure. Cytoplasmic proteins such as lactate dehydrogenase (LDH), damage-related molecular patterns (DAMPs) and cytokines including IL-1β, IL-18 were released from the swelling cells, and ultimately leading to inflammation (Shi et al., 2015). Subsequently, caspase-3, the executive-apoptotic protein, was found to have the property to cleave GSDME specifically after Asp270, destroying the self-inhibitory structure formed by the binding of N-terminal and C-terminal, resulting in a fragment known as the N-terminal domain of gasdermin E (GSDME-N), which penetrated the plasma membrane to transform slowly non-inflammatory apoptosis into rapidly inflammatory pyroptosis (Rogers et al., 2017; Wang et al., 2017). All members in the gasdermin family, except PJVK, have the gasdermin N-terminal with pore-forming activity and as such the ability to cause pyroptosis (Kovacs and Miao, 2017). Therefore, pyroptosis was redefined as a programmed cell death dependent on the gasdermin proteins in 2018 (Galluzzi et al., 2018).

As crucial substrates for pyroptotic cell death, GSDMD and GSDME mediated different types of pyroptosis. Most cases of GSDMD-dependent pyroptosis, except the direct cleavage of GSDMD by caspase-8, are inflammasome-dependent including canonical and non-canonical inflammasome-dependent pathways (Vanaja et al., 2015; Orning et al., 2018). GSDMD-mediated pyroptosis is usually accompanied by the assembly of inflammasomes, which induces caspase-1 dependent maturation of IL-1β and IL-18. These cytokines are in turn released through the pores formed by GSDMD, further amplifying the inflammatory response (He et al., 2015). Unlike GSDMD, GSDME-dependent pyroptosis is an inflammasome-independent pyroptosis without the assembly of inflammasomes. However, GSDME-N cleaved by caspase-3 has the pore-forming property to promote the release of cytokines such as IL-1β and IL-18, which have been confirmed in most pathological and pharmacological studies (Yu et al., 2019; Jiang et al., 2021; Xia et al., 2021). In addition, a recent study has indicated that the pores formed by GSDMD and GSDME not only act as channels for the release of IL-1β but also further activate NOD-like receptor protein 3 (NLRP3) inflammasome to promote IL-1β maturation (Zhou and Abbott, 2021). However, GSDME mediated an incomplete pyroptosis to release IL-1α but not IL-1β when caspase-1 was absent or inhibited (Aizawa et al., 2020). Similarly, the pore formation constructed by GSDME leads to cell swelling, disruption of cellular homeostasis and rupture of the plasma membrane. Ninjurin-1 (NINJ1), a conserved cell surface protein, has been proven to mediate the rupture of the plasma membrane and the subsequent release of DAMPs (Kayagaki et al., 2021). In contrast to small molecules cytokines that are released through GSDME-N formed membrane pores, most DAMPs such as high mobility group protein B1 (HMGB1) and LDH are passively released into the extracellular compartment after cell lysis to promote the inflammatory response.

Nevertheless, the activation of inflammatory caspases is not the only manner to activate the pore-forming activity of gasdermins. An investigation supported that natural killer (NK) cells cleave GSDME and induce pyroptosis in GSDME-expressing target cells, which is dependent on the release of cytotoxic granules. Granzyme B, released by NK cells, activates caspase-3 to enhance its lethality, as well as directly cleaves GSDME at the same residue to cause GSDME-dependent pyroptosis in tumors (Zhang Z. et al., 2020). Cancerous and normal cells are both capable of undergoing pyroptosis when stimulated *in vitro* or *in vivo*, as long as they express GSDME. Various scenarios as diverse as oxidative stress, intrinsic or extrinsic apoptotic pathways, endoplasmic reticulum stress and MAPK pathways that converge on the activation of caspase-3 in GSDME expressing cells can thereby result in GSDME-dependent pyroptosis (Wu et al., 2019; Yu et al., 2019; Zhang X. et al., 2020; Zhang et al., 2021a; Shangguan et al., 2021; Shen et al., 2021).

### The Crosstalk Between Apoptosis and GSDME-Mediated Pyroptosis

**FIGURE 1:**
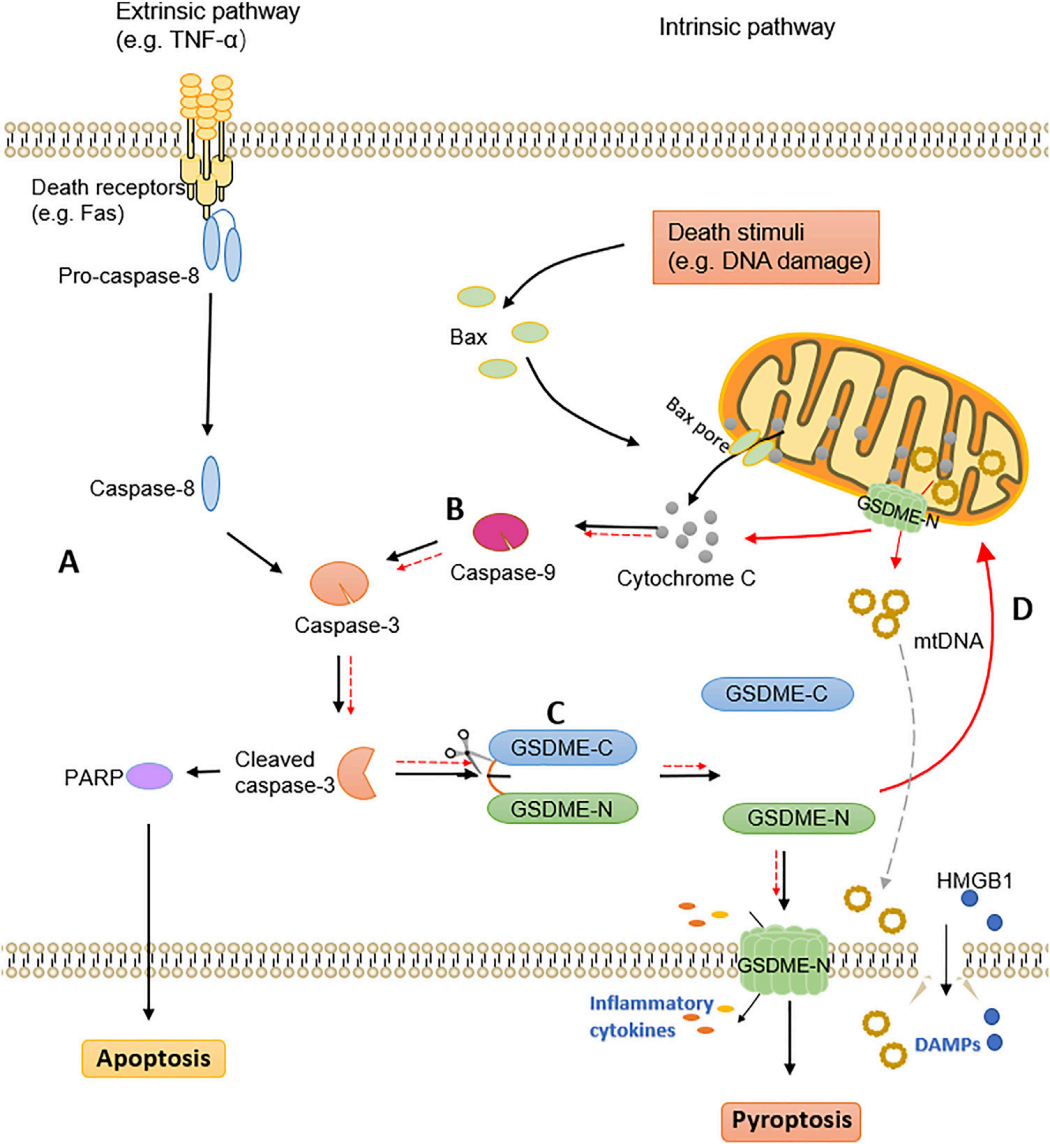
The crosstalk of apoptosis and GSDME-dependent pyroptosis. **(A)** The extrinsic pathway of apoptosis. **(B)** The intrinsic pathway of apoptosis. **(C)** The pyroptosis mediated by GSDME. Cleaved caspase-3 activates GSDME to generate GSDME-N fragments, which translocate to the plasma membrane and perforate to release inflammatory cytokines. DAMPs are released after cell rupture, ultimately leading to pyroptotic cell death. **(D)** GSDME-N targets mitochondrial membranes to form pores, releasing cytochrome C and mitochondrial DNA. Further activation of caspase-3 leads to self-amplification of pyroptosis.

The long-standing view of caspase-3 serving as a mediator of apoptosis has proven to be wrong by the discovery that GSDME is cleaved by caspase-3 and mediates pyroptosis. Actually, when the caspase-3 was activated in cells, GSDME expression determines the type of cell death, with apoptosis occurring at low levels and pyroptosis occurring at high levels. Exposing in cells with insufficient GSDME expression, tumor necrosis factor alpha (TNF-α) activates caspase-3 to cleave the apoptotic substrate protein poly ADP-ribose polymerase (PARP), triggering apoptotic cell death (Figure 1A). Inversely, in highly GSDME expressing cells, activation of caspase-3 preferentially cleaves GSDME, rather than PARP, to initiate pyroptosis preceding apoptosis (Figures 1B,C) (Wang et al., 2017). It needs to be mentioned that when the apoptotic cells are not effectively and timely removed by phagocytic cells, they will undergo secondary necrosis which is considered to be an unregulated procedure. Research has indicated that secondary necrosis occurs after induction of an apoptotic program in GSDME insufficient cells, which is distinct from GSDME-dependent apoptosis (Rogers et al., 2017). These observations further support that GSDME has a significant impact on regulating the mode of cell death.

Furthermore, both apoptotic and pyroptotic markers have been detected in lung cancer cells after chemotherapy, confirming the simultaneous occurrence and interaction of both processes (Lu et al., 2018). The inseparable relationship between GSDME-mediated pyroptosis and apoptosis has been confirmed by a large number of studies, with caspase-3 connecting these two modes of cell death. In addition to GSDME induced pyroptosis in the downstream of caspase-3, GSDME could also activate caspase-3 by functioning upstream of it, since GSDME-N targets mitochondria in apoptotic cells and permeates the mitochondrial membranes to release cytochrome C (Figure 1D) (Rogers et al., 2019). GSDME bridges the external and internal apoptotic pathways, as demonstrated by the cellular model of TNF-α-induced exogenous apoptotic pathway activation (Rogers et al., 2019). When GSDME-N targets mitochondrial membranes to form pores and further activate caspase-3, it will also cause self-amplification of pyroptosis. Another research further indicated that GSDME-N permeates mitochondria preferentially, and ruptures the plasma membrane later (Rogers and Alnemri, 2019). Mitochondrial DNA (mtDNA), as a DAMP, is released out of the mitochondria in the pore formation constructed by GSDME-N, and removed extracellularly upon rupture of the plasma membrane (de Torre-Minguela et al., 2021). Additionally, GSDME-N also induces mitochondrial network fragmentation during pyroptosis and apoptosis (de Torre-Minguela et al., 2021). Above all, as a novel mitochondrial pore-forming protein, GSDME is essential for the crosstalk between pyroptotic and apoptotic cell death pathways.

## The Role of GSDME in Diseases

### GSDME in Cancers

#### Tumor Suppression by GSDME

Back in 1998, a study first linked GSDME to cancer. In multiple estrogen receptor-positive breast cancer cell lines, low levels of GSDME were detected, linking the *gsdme* gene to cancer biology and indicating that it could be a determinant of hormonally unresponsive breast carcinomas (Thompson and Weigel, 1998). Subsequently, a series of studies suggested that it is a common phenomenon that GSDME expression is lower in most cancer cells than in normal cells due to the epigenetic inactivation caused by methylation (Akino et al., 2007; Kim et al., 2008a; Fujikane et al., 2010). It is well established that DNA methylation is one of the characteristic manifestations of cancer. The promoter of GSDME harbors CpG islands that regions enriched with CpG dinucleotides, which are prone to methylation in cancer cells. Methylation silences the *gsdme* gene, providing advantages for the growth of tumor cells (Kim et al., 2008a; Kim et al., 2008b). Therefore, the regulation of protein expression through methylation modification may be one of the important regulatory mechanisms for GSDME to participate in tumorigenesis.

Moreover, the tumor suppressive effect of GSDME has been confirmed by numerous *in vitro* and *in vivo* studies*.* Studies *in vitro* determined that the overexpression of GSDME significantly reduced the cell proliferation, colony formation and invasion capacity of cancer cells, whereas downregulation of GSDME significantly enhanced these activities (Akino et al., 2007; Kim et al., 2008a; Rogers et al., 2019). Compared to WT littermates, *gsdme* ^−/-^ mice exhibited significantly fewer and smaller tumors *in vivo* model of colitis-associated colorectal cancer (Tan et al., 2020). In another mice model of melanoma, tumor growth surveillance results showed that the rate of GSDME-KO tumors formation and growth reaching the sacrifice threshold was markedly faster than that of GSDME-expressing tumors (Rogers et al., 2019). Above, these findings demonstrated that GSDME might function as a tumor suppressor. In addition, the loss of function is attributed to cancer-associated GSDME mutations, which further supports the perspective of GSDME as a tumor suppressor (Zhang Z. et al., 2020).

**FIGURE 2:**
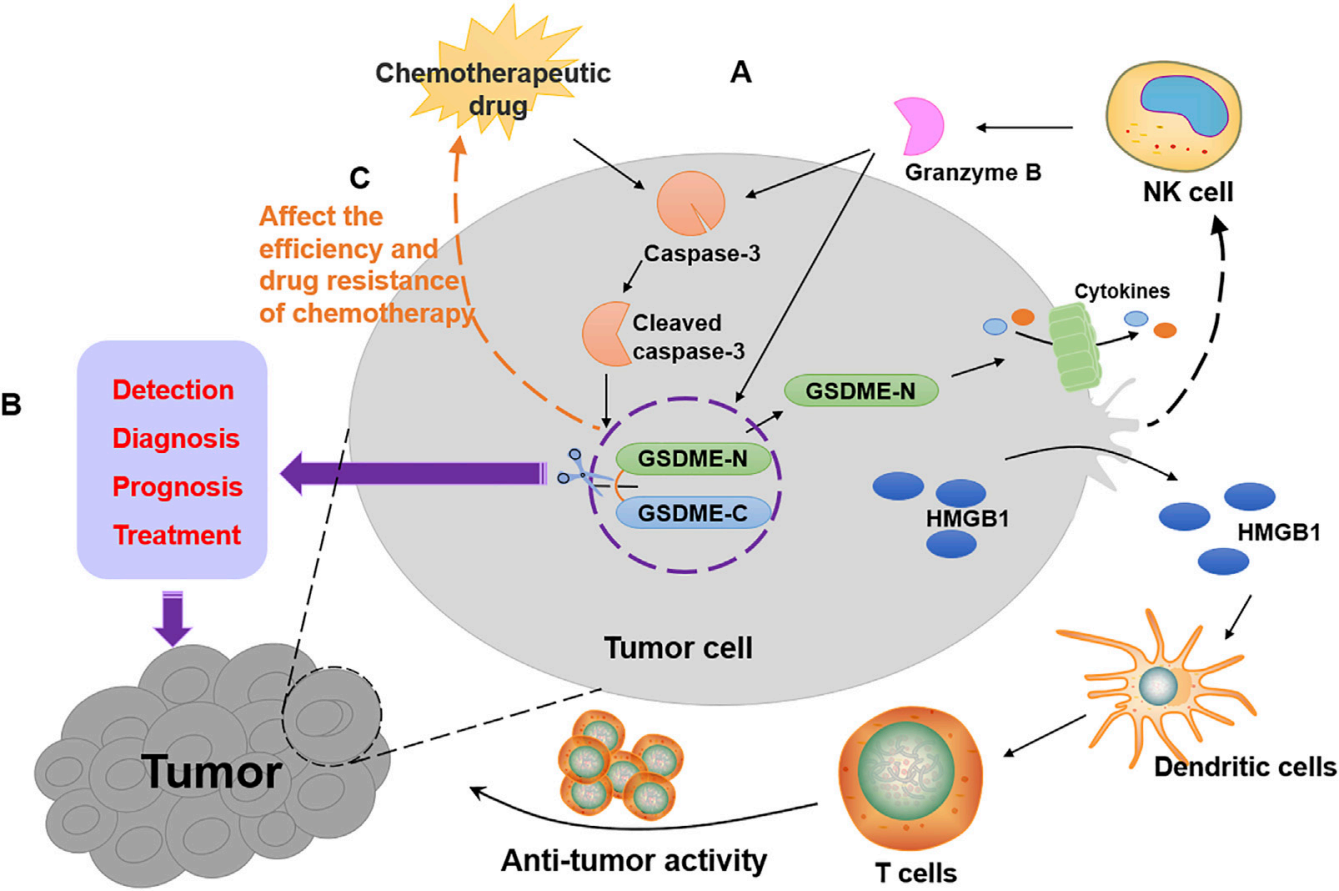
The role of GSDME in tumors. **(A)** The ways of tumor suppression by GSDME as shown by the black arrows in the figure. Chemotherapeutic drugs activate caspase-3 to form cleaved caspase-3, which leads to the cleavage of GSDME. The GSDME-N targets cell membrane to form pores, resulting in cancer pyroptotic cell death. Furthermore, granzyme B released by NK cells not only activates caspase-3 but also directly cleaves GSDME to cause pyroptosis in GSDME-expressing tumors, which enhances the function of tumor-infiltrating immune cells. Then, HMGB1 released by pyroptotic cell activates dendritic cells, which cause T cell proliferation to exert anti-tumor activity. **(B)** GSDME can be used for the detection, diagnosis, prognosis and treatment of cancer patients, as shown by the purple arrows in the figure. **(C)** The expression of GSDME affects the efficiency and drug resistance of chemotherapy, as shown by the orange arrows in the figure.

After clarifying GSDME function as a tumor suppressor, it is necessary to gain an understanding of how it plays its role in suppressing tumors. GSDME exerts its tumor suppressor effect in three main aspects (Figure 2A). On the one hand, GSDME-driven pyroptosis can be triggered by intrinsic stresses or extrinsic stimuli, such as chemotherapy and some molecular drugs, which activates caspase-3 to cleave GSDME, leading to inflammatory cell death (Wang et al., 2017; Lu et al., 2018; Yu et al., 2019). The mechanisms of cancer cell pyroptosis mediated by some chemotherapeutic drugs and compounds with anti-cancer activity are enumerated in Table 1. On the other hand, a positive correlation was found between GSDME expression and tumor-related macrophage phagocytosis as well as NK and CD8^+^T lymphocyte production and function (Zhang Z. et al., 2020). Granzyme B released by NK cells cleaves GSDME to trigger pyroptosis while enhancing the function of tumor-infiltrating immune cells to delay tumor growth furtherly (Zhang Z. et al., 2020). Besides, the combination treatment with v-raf murine sarcoma viral oncogene homolog B1 (BRAF) inhibitors and MEK inhibitors promotes GSDME cleavage and HMGB1 release in melanoma cells, which activate dendritic cells and ultimately lead to the proliferation of T cells to exert its anti-tumor effects (Erkes et al., 2020).

**TABLE 1:** Summary of mechanisms of activating GSDME-dependent pyroptosis.

	Molecules	Cancer types	Mechanism of activating GSDME dependent pyroptosis	References
Anticancer drugs	Paclitaxel and cisplatin	lung cancer	Caspase-3/GSDME	Zhang et al. (2019)
	Cisplatin and PLK1 inhibitor	oesophageal squamous cell carcinoma	caspase-8-mediated extrinsic apoptosis-BAX/caspase-3/GSDME	Wu et al. (2019)
	Lobaplatin	colon cancer	ROS/JNK/Bax-mitochondrial apoptotic pathway and caspase-3/-9 activation	Yu et al. (2019)
		cervical cancer	Caspase-3/GSDME	Chen et al. (2021a)
	Lobaplatin/birinapant	nasopharyngeal carcinoma cell	cIAP1/2/Ripoptosome (RIPK1/Caspase-8/FADD)/ROS/caspase-3/GSDME	Chen et al. (2020)
	TNFα+CHX/navitoclax	colorectal cancer	BAK/BAX-caspase-3-GSDME	Hu et al. (2020b)
	Oxaliplatin/GW4064	BAX/caspase-3/GSDME	Guo et al. (2021)
	Doxorubicin	breast cancer cells	ROS/JNK/caspase-3/GSDME	Zhang et al. (2021a)
Compounds with anticancer activity	Galangin	glioblastoma multiforme	Caspase-3/GSDME	Kong et al. (2019)
	Miltirone	hepatocellular carcinoma	ROS/MEK/ERK1/2-BAX-caspase 9-caspase 3-GSDME	Zhang et al. (2020b)
	Cannabidiol	hepatocellular carcinoma	ATF4/CHOP/caspase-3/GSDME	Shangguan et al. (2021)
	Triptolide	head and neck cancer	c-myc/HK-II-BAD/BAX/caspase-3-GSDME	Cai et al. (2021)
	Dihydroartemisinin	esophageal squamous cell carcinoma	PKM2-caspase-8/3-GSDME axis	Jiang et al. (2021)
	Dihydroartemisinin	breast cancer	AIM2/caspase-3/DFNA5 axis	Li et al. (2021d)
	Triclabendazole	breast cancer	ROS/JNK/Bax-mitochondrial apoptotic pathway-caspase-3-GSDME	Yan et al. (2021)
	Tetraarsenic hexoxide	triple-negative breast cancer	STAT3/ROS/caspase-3/GSDME	An et al. (2021)
	A novel 3′,5′-diprenylated chalcone	prostate cancer	PKCδ/JNK/Bax/caspase-3/GSDME	Zhang et al. (2020c)

ROS, reactive oxygen species; JNK, c-jun n-terminal kinase; cIAP1/2, cell inhibitor of apoptosis protein-1/2; RIPK1, receptor-interacting serine/threonine protein kinase 1; MEK, Mitogen-activated protein kinase Jun; ERK1/2, extracellular signal-regulated kinase 1/2; ATF4, activating transcription factor 4; CHOP, C/EBP homologous protein; HK-II, Hexokinase-II; PKM2, pyruvate kinase M2; AIM2, absent in melanoma 2; STAT3, signal transducer and activator of transcription 3; PKCδ, protein kinase Cdelta.

#### GSDME Methylation as a Cancer Biomarker

Based on the tumor inhibition property of GSDME and the prevalence of methylation in cancer, several studies have shown that GSDME methylation is a valuable molecular biomarker in cancer. The most common cancer among women is breast cancer, and some early detection and prognostic biomarkers are required for breast cancer diagnoses. TaqMan-methylation specific PCR was used to analyze the methylation of GSDME in tissues of breast cancer patients and non-cancer patients (Kim et al., 2008b). According to the results, GSDME methylation was observed in primary breast cancer tissues, and the receiver operating characteristic curves constructed by the relative level of GSDME methylation distinguishes the primary breast cancer tissue from normal tissue clearly, indicating that GSDME methylation has the potential to be a diagnostic marker for breast cancer. In addition, the study suggests that high GSDME methylation in breast cancer patients is linked to lymph node metastasis, therefore, breast cancer patients with the risk of lymph node metastasis can be identified by GSDME (Kim et al., 2008b). Research on analyzing methylation of CpGs at GSDME promoter regions in 123 primary breast adenocarcinomas and 24 healthy breast reductions demonstrated that the methylation of GSDME CpG4 can serve as an effective biomarker to detect breast cancer in the solid biopsy, indicating that GSDME methylation is an early event in breast cancer, which can be used as an indicator of early detection of breast cancer (Croes et al., 2017). Further analysis of survival data from breast cancer patients revealed an intimate correlation between GSDME methylation and 5-year overall survival (OS), suggesting that GSDME methylation has potential as a prognostic biomarker (Croes et al., 2018). Cancer is a diversified disease, but no biomarker can meet the requirements of diagnosis, prognosis and prediction simultaneously now. The above evidence shows that GSDME methylation appears to be an “ideal” tool on breast cancer screening, showing great potential in the early detection, diagnosis, metastasis organotropism and prognosis (Figure 2B).

In light of these results, further studies have found that the potential of GSDME biomarkers is not limited to breast cancer. A large-scale analysis of GSDME methylation in the largest cohort of colorectal cancer patients using public data from The Cancer Genome Atlas (TCGA) revealed that GSDME methylation was a possible biomarker for colorectal adenocarcinomas detection. By analyzing the TCGA methylation database, it was found that GSDME methylation might be prevalent in a variety of cancer types, which can be harnessed both as a “pan-cancer” biomarker and as a means to distinguish various cancers (Ibrahim et al., 2019). Furthermore, in addition to GSDME methylation, GSDME expression may also have prognostic potential. According to a pan-cancer analysis, GSDME expression was significantly associated with the survival prognosis of tumor patients (Zhang et al., 2021b). The poor prognosis of patients with hepatocellular carcinoma as well as head and neck squamous cell carcinomas have been proven to be highly correlated with the high expression of GSDME (Liu Z. et al., 2021; Fu and Song, 2021; Hu et al., 2021). Inversely, high GSDME expression is corresponding to a better prognosis in lung cancer and oral cancer (Huang et al., 2021; Wang et al., 2022). Currently, most biomarkers target only single cancer, and few biomarkers can detect and localize multiple cancer types simultaneously. The excellent expression and methylation properties of GSDME in diverse cancers make the *gsdme* gene an attractive target as cancer biomarkers.

#### GSDME as a Crucial Target in Chemotherapy

Upon treatment with chemotherapeutic drugs, GSDME is activated by caspase-3, resulting in pyroptosis of cancer cells with high GSDME expression (Wang et al., 2017). The relative mechanisms of GSDME activation are shown in Table 1. The pyroptosis induced by the cleavage of GSDME is the main form of cancer cell death. Cancer cells expressing GSDME are differently susceptible to pyroptosis induced by different chemotherapeutic agents. Paclitaxel and cisplatin, two representative chemotherapy drugs, were used to explore the different efficacy of pyroptosis in GSDME-expressing lung cancer A549 cells. Compared with paclitaxel, cisplatin elicited more obvious characteristics of pyroptosis, accompanied by higher production of activated caspase-3 and GSDME-N. GSDME knock-out in A549 cells significantly attenuated cisplatin-induced pyroptosis (Zhang et al., 2019). As an executioner of pyroptosis, GSDME contributes to the efficacy of chemotherapy. Analysis of the results of crystal violet staining *in vitro* found that GSDME deletion alleviated drug response and generated more drug-resistant persistence in lung cancer cells. Conversely, GSDME overexpression promoted the sensitivity of small-molecule inhibitors, which further confirms that GSDME-mediated pyroptosis is conducive to the drug response (Lu et al., 2018). Besides, decitabine, a hypomethylating agent reversing the silence of GSDME in cancers, is more effective in treating myelodysplastic syndrome when combined with chemotherapeutic agents, suggesting that there might be a positive correlation between GSDME expression and the effectiveness in anti-tumor of chemotherapy (Ball et al., 2017). Therefore, the high expression of GSDME often corresponds to the high efficiency of chemotherapy, indicating that GSDME might be a critical target for chemotherapeutic drugs (Figure 2C).

In the treatment of tumor patients, chemotherapeutic resistance of tumor cells is an inevitable problem that contributed to high mortality, and GSDME might play an important role in the generation of resistance to chemotherapy in cancer cells. The mRNA expression level of GSDME was significantly reduced in melanoma cells with 33-fold etoposide-resistant. The overexpression of the *gsdme* gene decreased etoposide-resistance by 30–35% compared to the normal group, which demonstrated that the reduction of GSDME mRNA level was associated with the increase of drug resistance (Lage et al., 2001). The chemosensitivity of esophageal squamous cell carcinoma cells was increased in cancer cells with high GSDME expression when treated with the combinations of cisplatin and PLK1 inhibitor BI2536 (Wu et al., 2019). Oxaliplatin, a platinum antitumor agent, is susceptible to drug resistance after repeated and long-term usage. Drug combination study suggests that compared with single-drug treatment, the combination of oxaliplatin and GW4064 can synergically inhibit colon cancer cells growth *in vitro* and slow down tumors growth *in vivo*. By activating Bax/caspase-3/GSDME pathway-mediated pyroptosis, GW3064 enhances the chemosensitivity of oxaliplatin (Guo et al., 2021). The enhancement of GSDME-dependent pyroptosis also corresponds to enhanced chemosensitivity in nasopharyngeal carcinoma and esophageal squamous cell carcinoma (Li Q. et al., 2020; Zheng et al., 2021). Thus, the activation of pyroptosis driven by GSDME may be an important manner to eliminate tumor resistance.

### GSDME in Non-Cancer Diseases

#### GSDME and Hearing Loss

Hearing loss (HL), a sensory impairment, is caused by mutations in different genes, including GSDME. Mutant GSDME, not the deficiency of GSDME, causes deafness, which is autosomal dominant, progressive, non-syndromic and sensorineural (Van Laer et al., 1998; Van Laer et al., 2005). Up to date, there are nine different mutations of GSDME related to HL, but all of them skipped the exon 8 during transcription, causing a frameshift and prematurely truncated protein, which destroyed the C-terminal domain, resulting in the loss of the self-inhibitory activity of GSDME as well as the emergence of cytotoxicity (Booth et al., 2018). It has been confirmed by the studies *in vitro* that cell mortality was increased after transfecting the mutation of GSDME (Gregan et al., 2003; Van Laer et al., 2004). The transfection experiments proved that HL related to GSDME is the result of a gain-of-function mutation. Previously study reported that apoptosis was activated in the outer hair cells of the cochlea in mice with age-related HL (Sha et al., 2009). The programmed cell death induced by mutant GSDME was apoptosis, no matter in the overexpression experiment or the physiological environment. Therefore, the possible pathogenesis of HL is that the mutant GSDME increases apoptosis of cochlear hair cells which are vital for hearing (Op de Beeck et al., 2011). A histopathological report, from the inner ear of a patient with HL related to GSDME variation, also displayed that the loss of inner and outer hair cells as well as the serious degeneration of stria vascularis and spiral ligament are the principal histopathologies of HL (Nadol et al., 2015). However, it is unclear whether pyroptosis is associated with HL induced by GSDME mutation.

#### GSDME-Dependent Pyroptosis Contributes to the Cytotoxicity of Chemotherapy

**FIGURE 3:**
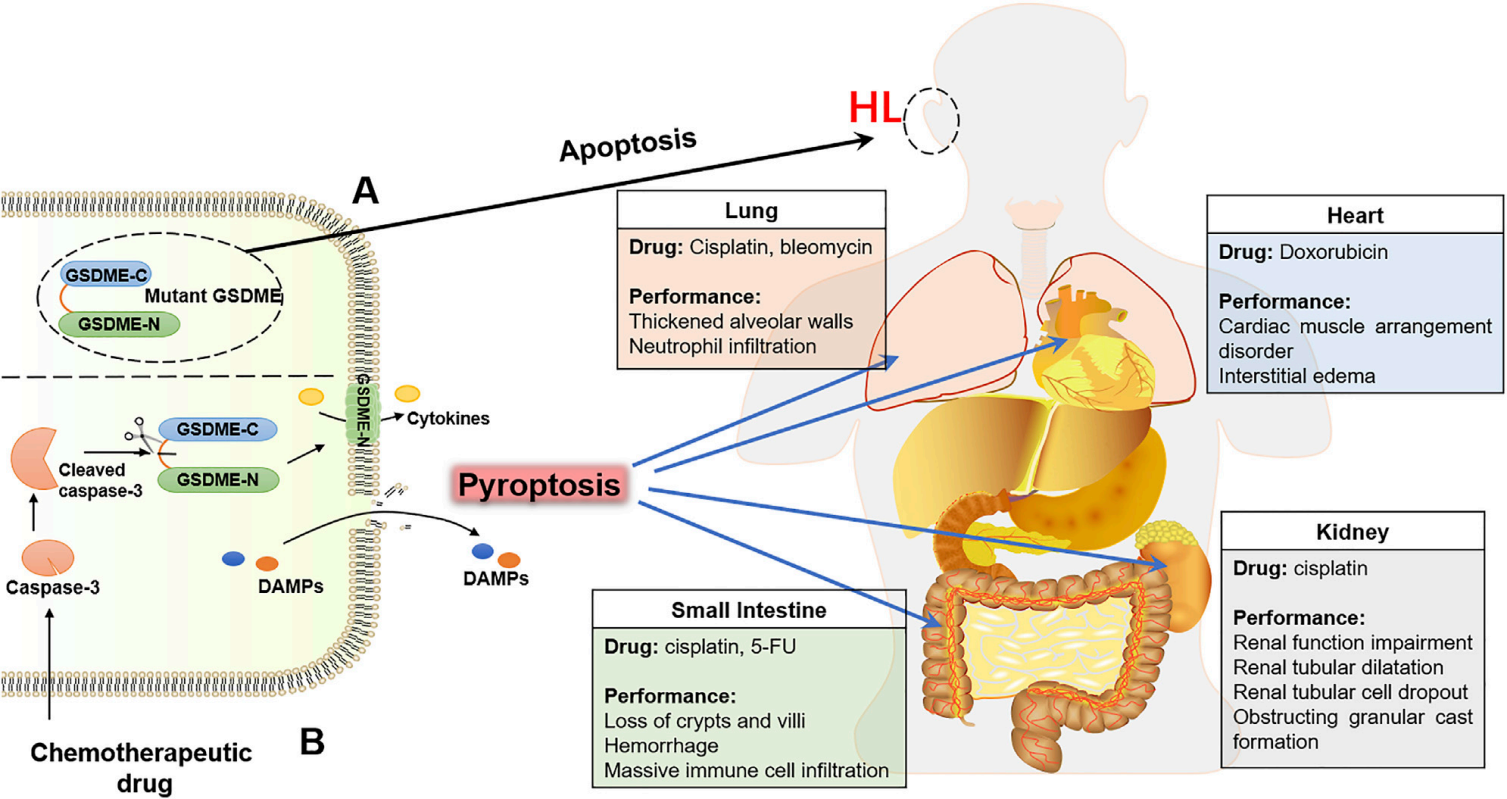
The GSDME contributes to the HL and toxicity of chemotherapy. **(A)** Mutant GSDME in connection with HL. **(B)** Chemotherapeutic drugs damage normal tissues by activating GSDME-mediated pyroptosis, including lung, heart, small-intestine and kidney.

As an effective method of tumor treatment, while exerting an anti-tumor effect, chemotherapeutic drugs always cause serious damage to healthy organs or tissues of cancer patients due to their indiscriminate cytotoxicity. The expression of the *gsdme* gene is higher in normal tissues than in tumor tissues, suggesting that the activation of GSDME may contribute to normal tissue damage during chemotherapy. It has been confirmed by several studies in recent years. Chemotherapeutic agents have been shown to damage normal human cells by activating GSDME-dependent pyroptosis, including human epidermal keratinocytes, human umbilical artery smooth muscle cells, and human placental epithelial cells. In addition, *gsdme* ^*−/−*^ mice were used to further confirm that GSDME was involved in chemotherapy adverse reactions, and the results showed that the chemotherapeutic drug cisplatin-induced alveolar wall thickening and destroyed the crypts and the villi, accompanied by immune cell infiltration in WT mice. Compared with WT mice, lung injury, small intestine injury and inflammation were improved after administering the chemotherapeutic drug in *gsdme* ^*−/−*^ mice (Wang et al., 2017). According to reports, one-third of cancer patients would experience nephrotoxicity caused by chemotherapeutic agents, which limited the dose of drugs and attenuated the antineoplastic effects. GSDME-dependent pyroptosis has been confirmed to contribute to chemotherapy-induced nephrotoxicity. The effects of cisplatin on GSDME-mediated pyroptosis were both time and dose-dependent in human renal tubular epithelial cells. The cleavage of GSDME was also detected in the mice model of cisplatin-induced nephrotoxicity, which examined decreased renal function, tubular dilatation, and serious death of renal tubular epithelial cells in comparison with the mice not given cisplatin (Shen et al., 2021). Additionally, it is further proved that GSDME deficiency significantly ameliorated cisplatin-induced renal damage and significantly improved renal function in virtue of GSDME-deficient mice (Xia et al., 2021). Doxorubicin (DOX) is an antineoplastic drug and its cardiotoxicity is the main adverse effect, which limited the clinical application of DOX. The serum and histopathological analysis of DOX-induced cardiotoxicity mice models revealed the severe cardiac injury, characterized by myocardial arrangement disorder and interstitial edema, along with a markedly increase in the expression of GSDME and N-terminal fragment. GSDME knockdown prevented DOX-induced pyroptosis of cardiomyocytes, hinting that GSDME was required for DOX-induced cardiomyocytes pyroptosis. Therefore, GSDME-mediated pyroptosis is inseparable from chemotherapy-induced cardiotoxicity (Zheng et al., 2020). PCR detection in normal tissues with cisplatin showed that the expression of GSDME-mRNA was upregulated, such as tongue, stomach, skin, kidneys and intestine. Cisplatin stimulated the cleavage of GSDME and led to pyroptotic cell death in normal tissue, which ultimately resulted in the side effects of chemotherapy (Huang et al., 2020). In conclusion, GSDME participates in the regulation of chemotherapy toxicities during chemotherapy (Figure 3B). In addition, a recent study has given evidence that GSDME-mediated pyroptosis is also engaged in the damaged intestine, stomach, liver and pancreas tissues caused by radiotherapy for colorectal cancer (Tan et al., 2022). Given this, how to balance the role of GSDME in tumor therapy and therapeutic toxicity is a challenge to be addressed when designing anti-tumor therapy regimens targeting GSDME.

#### GSDME-Mediated Pyroptosis in Kidney Diseases

**FIGURE 4:**
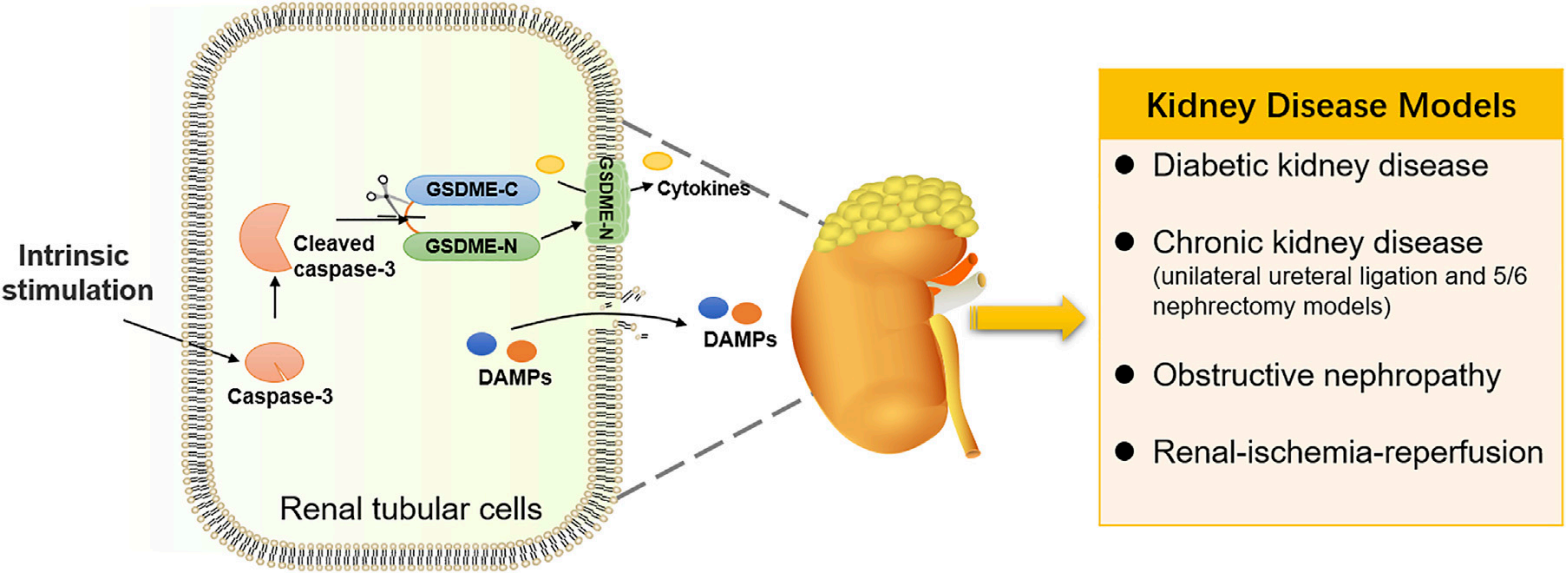
GSDME-mediated pyroptosis in kidney diseases. Intrinsic stimulation drives GSDME-mediated pyroptosis in renal tubular cells, participating in the occurrence and development of kidney diseases.

With advancements in research on pyroptosis, researchers have focused their attention on the GSDME-mediated pyroptosis in normal tissue injuries, especially kidney injuries. In addition to the chemotherapy-induced nephrotoxicity mentioned above, GSDME is also critical in regulating some nephropathies by promoting pyroptosis of normal renal tubular cells (Figure 4). As a complication of diabetes, diabetic kidney disease (DKD) is concerned with pyroptosis. GSDME levels were high in the tubular diabetic mice, and immunoblotting of the GSDME-N revealed aberrant activation of GSDME. In response to the treatment with caspase-3 inhibitor Z-DEVD-FMK, the kidney injury was decreased, coupled with the suppression of aberrant activation of GSDME. It was suggested that activation of GSDME plays a potentially critical role in DKD (Wen et al., 2020). A mini-review convincingly suggested that GSDME-mediated pyroptosis is a critical contributor to the pathogenesis and progression of DKD (Li W. et al., 2021). Furthermore, an elevated expression of GSDME-N was observed in a chronic kidney disease model. Fibrosis and inflammation of kidneys are restrained by expurgating the GSDME in the unilateral ureteral ligation and 5/6 nephrectomy models. In contrast, GSDME overexpression had an adverse effect on renal fibrosis. These evidences suggest that GSDME-mediated pyroptosis promoted inflammation to modulate renal fibrosis and renal dysfunction in chronic kidney disease (Wu et al., 2021). It was also confirmed in obstructive nephropathy. Upon ureteral obstruction, the activation of the TNF-α/caspase-3/GSDME pathway resulted in GSDME-mediated pyroptotic cell death, causing hydronephrosis and renal fibrosis, which ultimately promote the development of obstructive nephropathy (Li Y. et al., 2021). Moreover, severe kidney injury was observed in renal-ischemia-reperfusion (IR) model mice, whereas GSDME deficiency has a protective effect on kidney injury caused by IR (Xia et al., 2021). Thus, an increasing number of kidney diseases have been suggested to be related to GSDME and its dependent pyroptosis. Targeting GSDME for the treatment of nephropathy has a bright prospect in the future.

#### GSDME-Dependent Pyroptosis in Inflammatory Diseases

Inflammation can be triggered by pyroptosis, which has diametrically opposite effects on cancer cells and normal cells. In patients experiencing chemotherapy, pyroptosis triggers inflammation and prevents tumor cells from proliferating and migrating. However, dysregulation of pyroptosis, initiated by harmful stimuli, releases pro-inflammatory cytokines IL-1β and IL-18, leading to excessive inflammation and inflammation-related diseases. GSDME-mediated pyroptosis has been proven to trigger various inflammation reactions. CD147, a transmembrane protein, was increasing in the mucosa of inflammatory bowel disease (IBD) patients. Treatment with CD147 markedly enhanced the expression of GSDMD and GSDME proteins and activated pyroptosis to aggravate intestinal inflammation, suggesting that GSDME-mediated pyroptosis might be involved in the pathogenesis of IBD (Xu et al., 2020). Recently studies demonstrated that GSDME-mediated pyroptosis accelerated intestinal inflammation and contributed to the pathogenesis of Crohn’s disease (CD), a form of IBD (Tan et al., 2021). There was a significant presence of GSDME-N in the inflammatory colonic mucosa of patients with active CD. Besides, the pyroptosis mediated by GSDME contributed to mucosal inflammation in colitis mice induced by the 2,4,6-trinitrobenzenesulfonic acid via releasing HMGB1, which is a pro-inflammatory factor in intestinal epithelial cells (Tan et al., 2021). According to the current study, GSDME is present in human gingival epithelial cells similar to intestinal epithelial cells (HGECs). The gingival epithelial barrier can be destroyed by butyrate, which can up-regulate caspase-3 and GSDME, leading to pyroptosis of HGECs. The pyroptotic cells release pro-inflammatory mediators and induce periodontitis (Liu et al., 2019). By comparison with periodontitis lesions, the lesions of peri-implantitis are larger (Dionigi et al., 2020). The inflammation in peri-implantitis destroys the periodontal soft tissue and alveolar bone (Wilson et al., 2015). In inflamed gingival tissues of patients with peri-implantitis, caspase-3 and GSDME were increased and activated compared to healthy gingival tissues. And the axis of caspase-3/GSDME participated in HGECs pyroptosis induced by TNF-α (Chen C. et al., 2021). Therefore, GSDME-mediated pyroptosis is correlated to periodontal inflammation such as peri-implantitis and periodontitis. Meanwhile, the increased trimethylamine-N-oxide in periodontitis patients damages peripheral endothelial progenitor cells through pyroptosis driven by Bax/caspase-3/GSDME pathway, suggesting that GSDME activation may also be associated with endothelial dysfunction in periodontitis patients (Zhou et al., 2021). More recently, studies have certified that pyroptosis mediated by the caspase-3/GSDME axis is also concerned with inflammatory skin diseases (Liu J. et al., 2021), idiopathic inflammatory myopathies (Liu M. et al., 2020) and rheumatoid arthritis (Zhai et al., 2022). These researches demonstrated that GSDME has been identified as a potential therapeutic target for inflammation. However, since the role of pyroptosis in inflammatory disease has just begun to be comprehended, molecular or compounds targeting GSDME-mediated pyroptosis have not been reported for the treatment of inflammatory diseases. It is necessary to investigate the signaling pathways of GSDME-mediated pyroptosis furtherly, providing new therapeutic approaches for inflammatory diseases.

## The Development of Therapies Targeting GSDME

Due to the extensive interaction between caspases and gasdermins in the diseases, the inhibition of caspases activity by pan-caspase inhibitors has been used in experimental studies to significantly inhibit the gasdermin-mediated programmed cell death (Zhang J. et al., 2020; Alphonse et al., 2021; Li J. et al., 2021). However, there is an urgent need for specific targeted drugs to enter our vision as the extent of the role of the gasdermin protein varies between diseases. The discovery of the role of GSDME in various diseases reflects the fact that GSDME-targeted therapy might be an effective pattern for the treatments of various diseases, especially cancers. Herein, we summarize some therapeutic approaches targeting GSDME.

### Epigenetic Therapies Targeting GSDME

**FIGURE 5:**
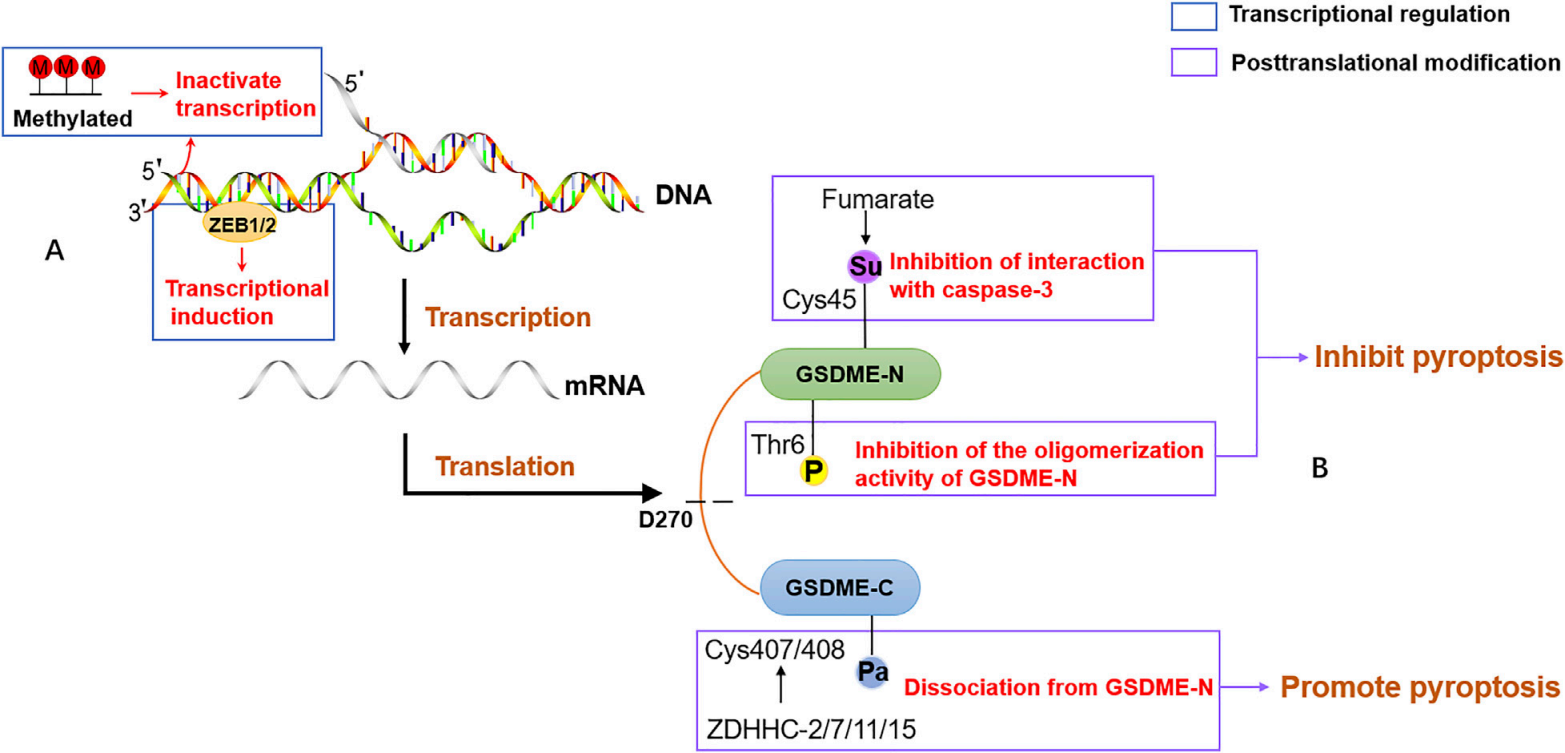
The epigenetic regulation of GSDME. **(A)** The transcriptional regulation of GSDME. The promoter of GSDME is methylated or binds to EMT-activating transcription factor ZEB1/2, leading to inactivate transcriptional and expression induction. **(B)** The posttranslational modification of GSDME. The phosphorylation of residue Thr6 in GSDME blocks the oligomerization activity of GSDME-N to prevent the formation of membrane pores. Fumarate promotes succinylation at Cys45 in GSDME and reduces the pore formation of the plasma membrane. ZDHHC proteins, including ZDHHC-2,7,11,15, palmitoylate GSDME at Cys407 and Cys408 to facilitate the separation of GSDME-C and GSDME-N.

A large amount of evidence indicates that there is an intimate connection between epigenetic regulations and cancer progression. As a vital player in cancers, it is urgent to explore the crucial epigenetic mechanism of GSDME. A recent study has given evidence that *gsdme* gene expression in different tumor models depends on epithelial-mesenchymal transition (EMT). ZEB1/2, a core EMT-activating transcription factor, directly binds to the promoter of the *gsdme* gene to drive transcription (Jia et al., 2021). Aberrant epigenetic features in cancer are often implicated in cancer pathogenesis. The epigenetic inactivation of GSDME in breast, colorectal, gastric and other cancers supported the view that GSDME is a tumor suppressor. Several epigenetic strategies are applied to avoid GSDME-evoked tumor cell suppression. DNA methylation, a critical modality of post-transcriptional modification, differs significantly between cancer and normal tissues. In addition to DNA methylation, posttranslational modification, another epigenetic mechanism, is also an important approach to regulate the activity of GSDME-driven pyroptosis. The phosphorylation of the highly conserved residue Thr6 in GSDME blocked the oligomerization activity of GSDME-N to prevent membrane leakage, which may play a crucial role in controlling pyroptotic activity (Rogers et al., 2019). *Plk1* is an oncogene that is abnormally elevated in a variety of tumors. The phosphorylation of GSDMA Thr8, corresponding to GSDME Thr6, is attributed to the PKL1 kinase activation, which possibly counteracts the function of GSDME as a tumor suppressor (Santamaria et al., 2011). Consistent with the Cys191 site of GSDMD, succination of GSDME at the Cys45 site was discovered to prevent the cleavage by caspase-3 and attenuate the GSDME-driven cell death (Humphries et al., 2020). Palmitoylation of GSDME also affected its pyroptosis. The C407 and C408 sites at the C-terminal of GSDME were palmitoylated to facilitate dissociation from N-terminal in a chemotherapy process, leading to increased pyroptosis in response to chemotherapeutic drugs (Hu, L. et al., 2020). However, there might be other epigenetic regulatory mechanisms in GSDME that have not been found yet up to now. The epigenetic regulations of GSDME are described in Figure 5.

Based on the epigenetic mechanism of GSDME, some epigenetic therapies have been devoted to enhancing antitumor efficacy. In cancer treatment, a strategy of combining DNA demethylation with chemotherapy has been established in a tumor-bearing mice model. Decitabine, a DNA methyltransferase inhibitor, was pretreated to remove GSDME methylation and promote its expression, and cisplatin was delivered to the tumor region by nanoliposomes to promote pyroptosis. This strategy was effective in improving the efficacy of chemotherapy and significantly reducing the recurrence of cancer (Fan et al., 2019). A poly porous microsphere, loaded with decitabine and doxorubicin, is applied to inhalation therapy of lung cancer by reversing *gsdme* silencing and promoting the induction of pyroptosis, which inhibits the growth and metastasis of lung tumors in tumor-bearing mice (Xie et al., 2021). Besides, treatment with such a microsphere generates an immune memory that provides continuous protection against neoplasm recurrence. 2-bromopalmitate, a palmitoylation inhibitor, transforms pyroptosis into apoptosis via inhibiting palmitoylation of the C-terminal of GSDME to decrease dissociation of the N-terminal, which is disadvantageous for chemotherapy (Hu L. et al., 2020). Therefore, promoting GSDME palmitoylation is a promising target for tumor therapy. Given the experimental research described above, GSDME-based epigenetic therapy might be a new approach for tumor treatment.

The epigenetic modification of GSDME appears to be manifested in the treatment of inflammatory diseases in addition to facilitating anti-tumor therapies. Fumarates such as dimethyl fumarate block pyroptosis by promoting succinate in human GSDMD at Cys191 and human GSDME at Cys45 to reduce inflammatory responses (Humphries et al., 2020). It is indicated that modification of the N-terminal of gasdermin proteins might be a drug target for resisting pyroptotic cell death. Moreover, disulfiram, a cysteine-modifying drug that prevents the formation of pores by modifying the Cys191 residue of human GSDMD, prevents the release of inflammatory factors and pyroptosis for the achievement of anti-inflammatory (Hu JJ. et al., 2020). Likewise, as an FDA-approved inhibitor of GSDMD pore formation, does disulfiram also target cysteine residues on GSDME-N? Consequently, similar inhibitors modifying the sites on GSDME-N to directly or indirectly inhibit the pore-forming activity and block pyroptosis will be one of the keys to targeting GSDME for the treatment of inflammatory diseases in the future. On the other hand, the discovery that the pore-forming activity of GSDMD-N is regulated by the Ragulator-Rag complex suggests that elucidating the regulatory mechanism of GSDME pore-forming activity also might be one of the future directions for blocking pyroptosis (Evavold et al., 2021).

### Therapies Targeting GSDME to Avoid Normal Tissue Destruction

Activation of GSDME exhibits excellent efficacy in tumor suppression but also damages normal tissues on account of deficient tumor targeting. Thus, how to balance the tumor therapy and normal tissues impairment is worth pursuing further. In addition to being activated by chemotherapeutic drugs, physical therapy such as cold atmospheric plasma (Yang et al., 2020), photodynamic therapy (Li L. et al., 2021) and ionizing radiation therapy (Su et al., 2021) have also been shown to play an anticancer role through GSDME. Photodynamic therapy, which destroys tumor cells via taking advantage of photosensitizer and visible light to produce ROS, is superior in less toxic to normal tissues compared to chemotherapy (Dobson et al., 2018). Besides, a universal tumor-targeting nanoliposome has been widely used in clinical trials, which can deliver drugs to tumor tissues and accurately achieve the chemotherapeutic pyroptosis of tumor cells to alleviate normal tissue damage (Fan et al., 2019). Considering the high efficacy of specific targeted therapy and the reduction of adverse reactions simultaneously, the targeted drugs incorporated chemotherapeutic drugs and photosensitizers are developed. MCPP, a nanoparticle synthesized with chemotherapeutic drugs paclitaxel and photosensitizers photosensitizer purpurin 18 (P18), exhibited robust induction of GSDME-dependent pyroptosis and fewer destruction of normal tissues. Mechanistically, GSDME-mediated pyroptosis is synergically driven by chemo-photodynamic therapy and controlled-release paclitaxel, releasing DAMPs and triggering immune responses, which plays an anti-tumor role and generate immunological memory to prevent tumor recurrence (Xiao et al., 2021). A biomimetic nanoparticle (BNP), combined with chemotherapy and phototherapy similarly, is applied to cure solid tumors with minimal systemic toxicity. Loading with indocyanine and decitabine, BNPs are aggregated at solid tumor sites, then induce the activation of caspase-3 by photo-activation and release decitabine to upregulate the expression of GSDME, resulting in the cleavage of GSDME, which triggers pyroptosis of tumor cells to activate anti-tumor immunity (Zhao et al., 2020). Both MCPP and BNP specifically target tumor tissue, showing powerful anti-tumor efficacy and minimal normal tissue damage. Therefore, therapies targeting GSDME harbor great prospects in enhancing anti-tumor efficacy and reducing toxic and side effects.

## Conclusion and Perspectives

GSDME, as a non-syndromic hearing loss gene, was first discovered in 1995 (Van Camp et al., 1995) and classified as a member of the gasdermin family in 2007 (Tamura et al., 2007). The connection between GSDME and cancer was well established but its specific pathological mechanisms were still unclear until 2017 when Wang et al. discovered GSDME-mediated pyroptosis, opening a new chapter in GSDME exploration. In the subsequent studies, GSDME was considered as a tumor suppressor and tumor biomarker. As a tumor biomarker, GSDME methylation shows great promise for early detection, diagnosis, prognosis and treatment in cancers. In cancer therapy, the tumor suppression of GSDME is achieved by the cancer cell pyroptosis and activation of immune responses. GSDME-dependent pyroptosis, activated by chemotherapeutic drugs and physical stimuli, has been suggested to have the property of killing tumor cells. As a “switch”, caspase-3 converts apoptosis into pyroptosis in GSDME-expressing cells. Actually, in the process of chemotherapy, both of them coexist, and if the apoptotic cells cannot be cleared timely, secondary death will occur in the late stage. GSDME expression determines the pattern of programmed cell death, but it is unclear how the high expression of GSDME completely overrides apoptosis.

Regardless of cancer cells or normal cells, GSDME-mediated pyroptosis is closely associated with mitochondrial apoptosis. The pore-forming activity of GSDME-N extends beyond the cell membrane to the mitochondrial membrane, which permeabilizes mitochondria to release cytochrome C and mtDNA (Rogers et al., 2019; de Torre-Minguela et al., 2021). The crosstalk between GDSME and GSDMD, which are the crucial proteins in the gasdermin family mediating pyroptosis, deserves attention. Similarly, the GSDMD-N terminal also contributes to mitochondrial permeability, releasing cytochrome C and activating caspase-3. The upstream inflammasomes of GSDMD may also affect the process of GSDME-mediated pyroptosis. AIM2 inflammasome induces GSDME expression through the activation of caspase-3 in breast cancer cells (Li Y. et al., 2021). While in zebrafish models, NLRP3 inflammasome induced pyroptosis in a GSDME-dependent manner independent of apoptosis-associated speck-like protein containing a caspase recruitment domain (ASC) (Li JY. et al., 2020). The crosstalk between GSDMD and GSDME-driven pyroptosis reflects the significance of pyroptosis in disease.

Encouraged by the tumor suppressive effects of GSDME, several strategies have been developed to target GSDME for cancer therapy. Based on the epigenetic mechanism of GSDME, especially DNA methylation, epigenetic therapy of GSDME is widely used as an adjuvant in tumor combination therapy to improve anti-tumor efficacy. It should be aware that the process of tumor therapy is often accompanied by many adverse reactions. A significant role for GSDME in chemotherapy-related side effects cannot be ignored. Compared with traditional caspase-3 inhibitors Ac-DMPD-CMK and Ac-DMLD-CMK, GSDME-derived caspase-3 inhibitors, exhibit lower IC_50_ which alleviates acute hepatic failure by inhibiting apoptosis and pyroptosis (Xu et al., 2021). In addition, some therapies target GSDME to avoid normal tissue destruction. The nanoparticles loaded with chemotherapeutic agents and photosensitizers are particularly capable of delivering drugs to the tumor tissues, which have strong advantages in high efficiency and low toxicity in cancer therapy. However, the limitations of targeted therapies, such as drug resistance and exorbitant price, suggest the importance of exploring alternative therapies that can reduce side effects while ensuring efficacy. As previously mentioned, GSDME has been implicated in the pathogenesis of inflammatory diseases, especially kidney diseases, but there is a great deal of room to research the GSDME-targeted therapies to block pyroptosis and alleviate inflammatory response. A recent preprint mentions that GSDME also plays an important role in Corona Virus Disease 2019 (COVID19) (Planès, et al., 2022, preprint). The activation of the caspase-3/GSDME axis induces airway epithelial cell death during SARS-CoV-2 infection when GSDMD is deficient (Planès, et al., 2022, preprint). It suggests that GSDME has the potential to be a marker for the severity of COVID19. The universality of GSDME in human disease reveals its great potential for targeted therapies in the future.

The bright prospects of GSDME-targeted therapies are circumscribed by the current incomplete disclosure of the structure and pyroptotic regulatory mechanism of GSDME. Recent studies on the mechanism of the gasdermins activation by protease may provide several directions for further research on GSDME. Two studies of GSDMD recognition at the exosite by inflammatory caspases suggest that gasdermins may provide a critical site for binding to the protease independent of the cleavage site (Liu Z. et al., 2020; Wang et al., 2020). Distinct from GSDMD, caspase-3 and granzyme B bind GSDME mainly through the cleavage site sequence and cleave human or mouse GSDME directly at residue D270, and the contribution of their external site is unknown because of the undiscovered crystal structure of GSDME. Therefore, further biological studies on GSDME should focus on the analysis of its structure and biological pathways, which are conducive to the development of drugs in the future and may provide directions for disease therapies targeting GSDME.

## Glossary

ASC: Apoptosis-associated speck-like protein containing a caspase recruitment domain
BRAF: V-raf murine sarcoma viral oncogene homolog B1
CD: Crohn’s disease
COVID19: Corona Virus Disease 2019
DAMPs: Damage-related molecular patterns
Dex: Dexamethasone
DFNA5: Deafness autosomal dominant 5
DKD: Diabetic kidney disease
DOX: Doxorubicin
EMT: Epithelial-mesenchymal transition
GEPIA2: Gene Expression Profiling Interactive Analysis 2
GSDMA: Gasdermin A
GSDMB: Gasdermin B
GSDMC: Gasdermin C
GSDMD: Gasdermin D
GSDME: Gasdermin E
GSDME-C: Gasdermin E C-terminal domain
GSDME-N: Gasdermin E N-terminal domain
HGECs: Human gingival epithelial cells
HL: Hearing loss
HMGB1: High mobility group protein B1
IBD: Inflammatory bowel disease
IL-18: Interleukin-18
IL-1β: Interleukin-1β
IR: Ischemia-reperfusion
KO: Knockout
LDH: Lactate dehydrogenase
MEK: Mitogen-activated protein kinase Dax
mtDNA: Mitochondrial DNA
NK: Natural killer
NLPR3: NOD-like receptor protein 3
OS: Overall survival
PARP: Poly ADP-ribose polymerase
PCD: Programmed cell death
PJVK: Pejvakin
PLK1: Polo-like Kinase 1
TCGA: The Cancer Genome Atlas
TNF-α: Tumor necrosis factor alpha
WT: Wild type
